# Potential association between IL-17A inhibition and blood pressure reduction in patients with axial spondyloarthritis

**DOI:** 10.3389/fimmu.2026.1826954

**Published:** 2026-06-22

**Authors:** Qianqian Wu, Yifan Wang, Ting Zhao, Jie Guo, Xiaomin Zhang, Yang Tu, Feng Wang

**Affiliations:** Department of Rheumatology, Immunology and Allergy, Shanghai General Hospital, Shanghai Jiao Tong University School of Medicine, Shanghai, China

**Keywords:** hypertension, ax-SpA, blood pressure, IL-17A, IL-17A inhibition

## Abstract

**Objective:**

This study aimed to investigate longitudinal changes in blood pressure associated with IL-17A inhibition in patients with axial spondyloarthritis (ax-SpA).

**Methods:**

This was a single-center, prospective, single-arm exploratory study that included 50 axSpA patients who received the IL-17A inhibitor Ixekizumab (160 mg loading dose at week 0, followed by 80 mg every 4 weeks for 12 weeks). Disease activity (ASDAS-CRP, ASDAS-ESR, BASDAI), inflammatory markers (CRP, ESR, IL-6), and blood pressure were monitored at baseline and every 4 weeks after treatment initiation. A linear mixed-effects model was used to analyze longitudinal changes in blood pressure.

**Results:**

IL-17A inhibition significantly improved disease activity and reduced inflammatory marker levels (all *p* < 0.001). Systolic blood pressure (SBP), diastolic blood pressure (DBP), and mean arterial pressure (MAP) all showed time-dependent decreases. Linear mixed-effects models estimated a reduction of 0.90 mmHg every 4 weeks for SBP (95% CI: -1.87 to 0.07, *p* = 0.071) and 0.91 mmHg per month for DBP (95% CI: -1.64 to -0.17, *p =* 0.016). Subgroup analysis showed that the rate of DBP decline was more pronounced in patients with baseline hypertension (n=36). After 12 weeks, SBP and DBP were decreased by 4.80 mmHg and 5.10 mmHg, respectively. No obvious side effects were observed in hematological, hepatic or renal function tests (both *p* < 0.01).

**Conclusion:**

In this exploratory pilot study, IL-17A inhibition was associated with effective control of disease activity and a concomitant, time-dependent reduction in blood pressure in patients with ax-SpA. Linear mixed-model analysis suggested this reduction was particularly evident for DBP in patients with baseline hypertension. These findings provide preliminary clinical evidence supporting further investigation into the potential cardiovascular modulatory effects of IL-17A-targeted therapy. However, the uncontrolled design necessitates confirmation in randomized trials.

## Highlights

- IL-17A reduction in SBP and DBP in patients with axSpA.
- Linear mixed-effects models quantified the reduction at 0.90 and 0.91 mmHg every 4 weeks for SBP and DBP, respectively.
- Patients with baseline hypertension exhibited a more pronounced rate of DBP reduction in the mixed model, suggesting a potential differential effect that needs further study.

## 1 Introduction

Axial spondyloarthritis (ax-SpA) is a chronic inflammatory disease primarily affecting the axial skeleton, leading to sacroiliitis and progressive structural damage. The condition typically presents with intermittent chronic inflammatory back pain and progressive restriction of spinal mobility (1).

While the application of biological agents has significantly improved patient prognosis in recent years, persistent systemic inflammation can still contribute to multisystem complications (2, 3). Notably, patients with AS exhibit a markedly increased cardiovascular risk, with hypertension being a major modifiable risk factor. A meta-analysis of 36 studies involving 119,427 AS patients reported an overall hypertension prevalence of 23% in this population (4). In a study from Greece, 30% of patients with axial spondyloarthritis (axSpA) had comorbid hypertension (5). Other studies have shown that 34% of AS patients exhibit abnormal blood pressure, with hypertension identified as the sole independent risk factor significantly associated with cardiovascular target organ damage (6).

Longitudinal evidence suggests that over a 10-year follow-up, patients with AS achieved significantly lower rates of adequate cardiovascular risk control compared to healthy controls (37% vs. 54%), despite a similar prevalence of hypertension at baseline (7). These findings underscore the clinical challenges in blood pressure management for this population.

The pathogenesis of elevated blood pressure in autoimmune diseases is multifactorial and is not limited to mechanical injury of the arterial wall. Chronic inflammation and immune-mediated mechanisms play a pivotal role in blood pressure dysregulation in patients with systemic autoimmune diseases (8). Specifically, chronic inflammation activates innate and adaptive immune responses, induces endothelial dysfunction, promotes vascular remodeling, and stimulates the renin-angiotensin system, thereby disrupting normal blood pressure homeostasis. Furthermore, immune cell dysregulation, along with excessive release of pro-inflammatory cytokines interleukin-6 (IL-6), tumor necrosis factor-α (TNF-α) and IL-17A, is closely associated with the onset and progression of hypertension in rheumatic diseases. The abnormal activation of Th17 cells and their signature cytokine IL-17A represents a shared nexus between AS and hypertension. Studies demonstrate that IL-17A participates in blood pressure regulation through mechanisms including induction of endothelial dysfunction, promotion of vascular remodeling, and sodium retention (9, 10), providing molecular insight into immune-cardiovascular interactions. These findings provide a strong theoretical basis for exploring the potential impact of anti-inflammatory therapies on blood pressure regulation. In clinical practice, IL-17A monoclonal antibodies such as secukinumab have emerged as significant therapeutic breakthroughs in AS treatment, effectively suppressing inflammatory activity and delaying radiographic progression (11). Real world studies have further confirmed the effectiveness of IL-17A inhibition in reducing disease activity and improving patient-reported outcomes in axial spondyloarthritis (12). However, the potential effect of IL-17A-targeted therapy on blood pressure dynamics in patients with AS remains unclear. This exploratory study aims to provide preliminary clinical evidence by investigating the potential effects of IL-17A inhibition on blood pressure in patients with ax-SpA.

## 2 Methods

### 2.1 Study design and participants

This single-center, prospective, single-arm, exploratory study enrolled 50 consecutive patients (30 males; 20 females; mean age 39.3 ± 15.5 years) with ax-SpA who received the interleukin-17A (IL-17A) inhibitor Ixekizumab (Eli Lilly, USA) at the Department of Rheumatology and Immunology of Shanghai General Hospital between April 2024 and March 2025. All participants fulfilled the 2009 Assessment of SpondyloArthritis International Society (ASAS) classification criteria for axSpA (13).

### 2.2 Diagnostic criteria

Diagnosis was based on the 2009 ASAS classification criteria for ax-SpA: 1) Chronic back pain (≥3 months duration) with onset ≤45 years; 2) Either Imaging evidence of sacroiliitis plus ≥1 SpA feature or HLA-B27 positivity with ≥2 SpA features.

### 2.3 Inclusion criteria

Participants met all following requirements:

1. ASAS-confirmed axSpA diagnosis;
2. Active disease defined by BASDAI ≥4 (14), or ASDAS ≥2.1 (15);
3. Age ≥18 years;
4. Provided signed informed consent.

### 2.4 Exclusion criteria

Key exclusion criteria included: active infections (bacterial/viral/fungal), tuberculosis infection, flaring inflammatory bowel disease, and malignancy (current or history).

### 2.5 Data acquisition protocol

Comprehensive baseline profiling included Demographics: gender, age, disease duration, comorbidities, and medication history; Clinical indicators: blood pressure, complete blood count, C-reactive protein (CRP), erythrocyte sedimentation rate (ESR), interleukin-6 (IL-6), liver and kidney function tests (ALT, AST, BUN, Scr).

### 2.6 Therapeutic intervention and outcome measures

The protocol consisted of: A) Ixekizumab treatment: Patients received a subcutaneous loading dose of 160 mg at week 0, followed by 80 mg every 4 weeks for 12 weeks. B) Discontinuation criteria: Treatment was ceased for disease progression (defined as a BASDAI score increase ≥2) or occurrence of ≥Grade 3 adverse events per CTCAE v6.0 guidelines. C) Comprehensive assessments included: (1) Blood pressure monitoring every 4 weeks; (2) disease activity evaluation using BASDAI (12) and ASDAS-CRP (15); (3) laboratory tests (complete blood count, hepatic/renal function); (4) safety monitoring documenting adverse drug reactions and major adverse cardiovascular events.

### 2.7 Hemodynamic assessment protocol

Blood pressure was measured according to American Heart Association (AHA) guidelines (16). After ≥5 minutes of seated rest, three consecutive measurements were taken on the upper arm using a validated automated oscillometric device (OMRON HEM-7322, Omron Healthcare, Kyoto, Japan), with one-minute interval between readings. The average of the last two readings was recorded for analysis.

### 2.8 Statistical analysis

Given the exploratory nature, the statistical analysis plan was not prospectively registered. Analyses were performed using R software (version 4.3.0). Continuous variables were presented as mean ± standard deviation or median (interquartile range); categorical variables as frequency (percentage). Baseline characteristics were summarized descriptively. Paired t-tests were used to evaluate disease activity indices and inflammatory markers before and after 12 weeks of treatment.

The primary analysis for blood pressure was performed using linear mixed-effects models (LMMs) fitted using the lme4 (v1.1-35) and lmerTest (v3.1-3) packages. Separate models were constructed for SBP and DBP. Fixed effects included time (continuous, every 4 weeks), age, gender, antihypertensive drug use, nonsteroidal anti-inflammatory drug (NSAID) use, and baseline hypertension status (SBP ≥130 mmHg or DBP ≥80 mmHg). A random intercept for each subject was included. The interaction between time and baseline hypertension status was assessed using likelihood ratio tests. Sensitivity analyses redefined hypertension using ACC/AHA (*>*130/80 mmHg) and ESC/ESH (*>*140/90 mmHg) thresholds (16). Model explanatory power was evaluated using marginal and conditional R² (MuMIn package v1.47.5). All tests were two-tailed, with *p* < 0.05 considered significant.

## 3 Results

### 3.1 Clinical characteristics

**Figure 1:**
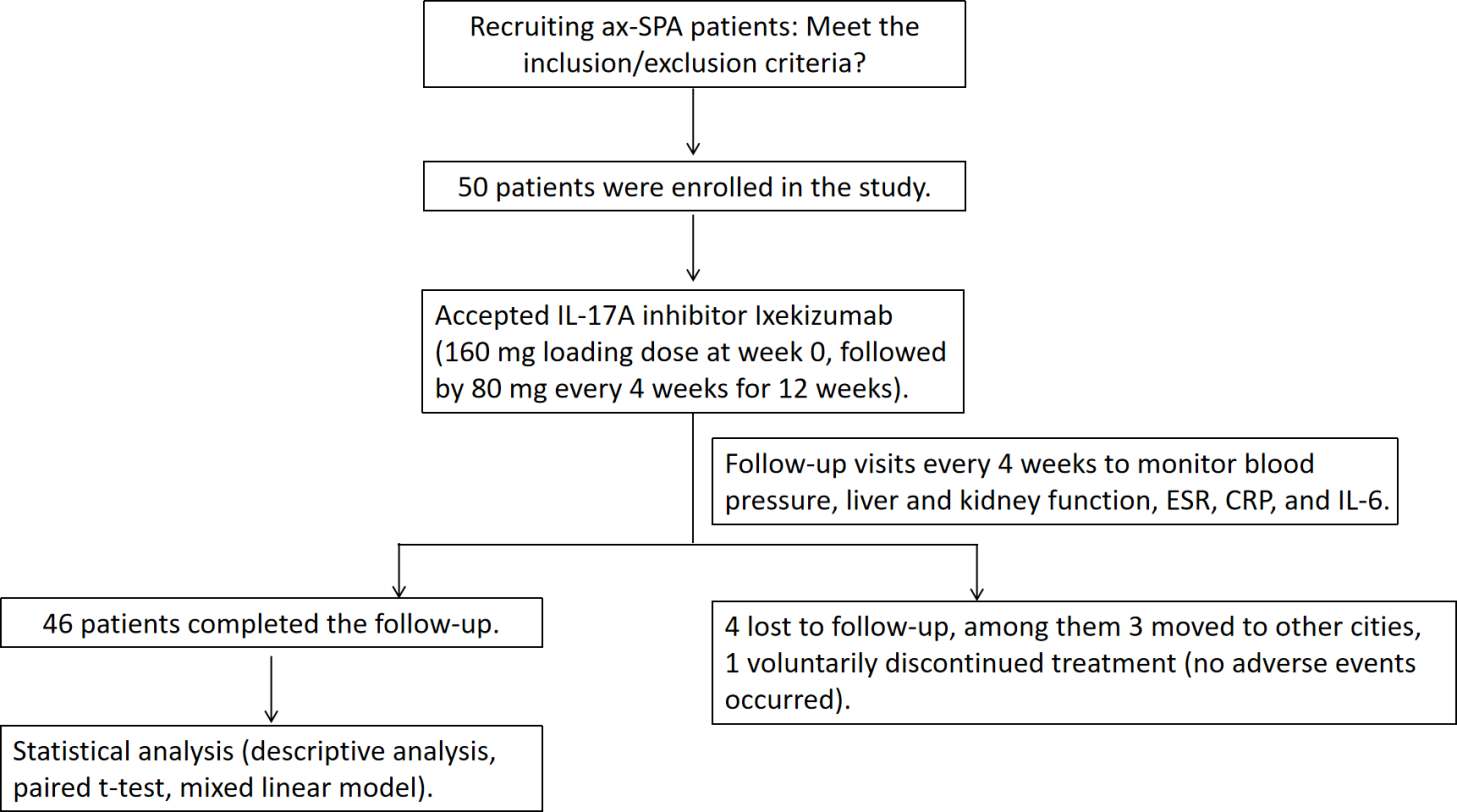
Research flowchart.

The flowchart was shown in **Figure 1**. A total of 50 patients were recruited, with 46 (92%) completing the 12 weeks follow-up.

Baseline characteristics are shown in **Table 1**. The cohort had a mean age of 39.3 years, was 60% male, and had a mean disease duration of 48.4 months. HLA-B27 positivity was 84% and the prevalence of hypertension was 72%. Other comorbidities included diabetes (4%), hyperlipidemia (28%), cardiovascular disease (4%), chronic nephritis (2%), hypothyroidism (2%), eye inflammation (6%), and atopic reactions (6%). Prior to biologic treatment, 84% received NSAIDs, 42% sulfasalazine (SASP), and 6% methotrexate (MTX).

**Table 1:** Clinical characteristics of AS patients.

Characteristics	Value
n	50
age, mean(SD)	39. 3(15. 5)
male, n(%)	30(60%)
duration(month), mean(SD)	48. 4(68. 7)
HLA-B27+, n(%)	42(84%)
hypertension, n(%)	36(72%)
ACEI or ARB	6(16. 7%)
CCB	2(5. 5%)
Diabetes, n(%)	2(4%)
Hyperlipidemia, n(%)	14(28%)
Cardiovascular disease, n(%)	2(4%)
Chronic nephritis, n(%)	1(2%)
Hypothyroidism, n(%)	1(2%)
Eye inflammation (uveitis or iridocyclitis), n(%)	3(6%)
Atopic reaction, n(%)	3(6%)
Hepatitis B, n(%)	1(2%)
Hepatitis C, n(%)	0
tuberculosis, n(%)	0
Previous nonbiological treatment	
Methotrexate, n(%)	3(6%)
Sulfasalazine, n(%)	21(42%)
Nonsteroidal anti-inflammatory drugs, n(%)	42(84%)

Values are shown as the median or the number of patients (the percentages). SD, Standard Deviation; ACEI, Angiotensin-Converting Enzyme Inhibitors; ARB, Angiotensin Receptor Blockers; CCB, Calcium Channel Blockers.

### 3.2 Clinical effectiveness of IL-17A inhibition

**Figure 2:**
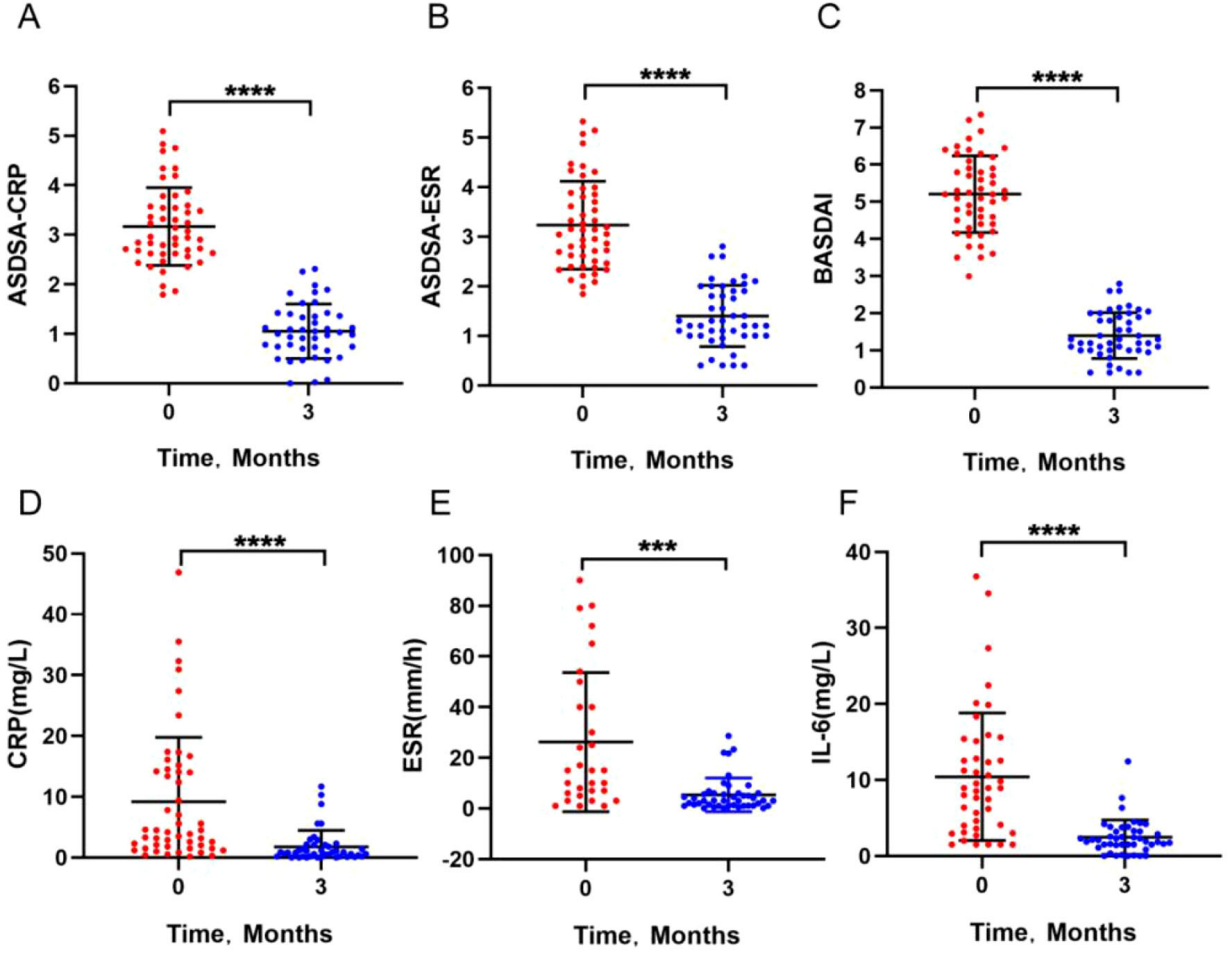
Clinical effectiveness of IL-17A inhibition at baseline and after 12 weeks **(A)** ASDAS-CRP, **(B)** ASDAS-ESR, **(C)** BASDAI, **(D)** CRP, **(E)** ESR, **(F)** IL-6. Data are shown as individual points with median and interquartile range (IQR). ***p < 0.001, ****p < 0.0001 (paired t-test).

IL-17A inhibition led to significant disease control. After 12 weeks, the Ankylosing Spondylitis Disease Activity Score (ASDAS) decreased significantly, including ASDAS-CRP from 3.17 ± 0.79 to 1.05 ± 0.55 (*p* < 0.001; **Figure 2A**) and ASDAS-ESR from 3.23 ± 0.89 to 1.40 ± 0.62 (*p* < 0.001; **Figure 2B**) consistently, Bath Ankylosing Spondylitis Disease Activity Index (BASDAI) from 5.21 ± 1.03 to 1.40 ± 0.62 (*p* < 0.001; **Figure 2C**). Inflammatory levels were effectively reduced, including CRP decreased from 9.19 ± 10.60 mg/L to 1.79 ± 2.68 mg/L (p = 0.001; **Figure 2D**), ESR from 26.20 ± 27.41 mm/h to 5.40 ± 6.34 mm/h (*p* < 0.001; **Figure 2E**), and IL-6 from 10.42 ± 8.38 pg/mL to 2.48 ± 2.27 pg/mL (*p* < 0.001; **Figure 2F**).

### 3.3 Safety of IL-17A inhibition

**Figure 3:**
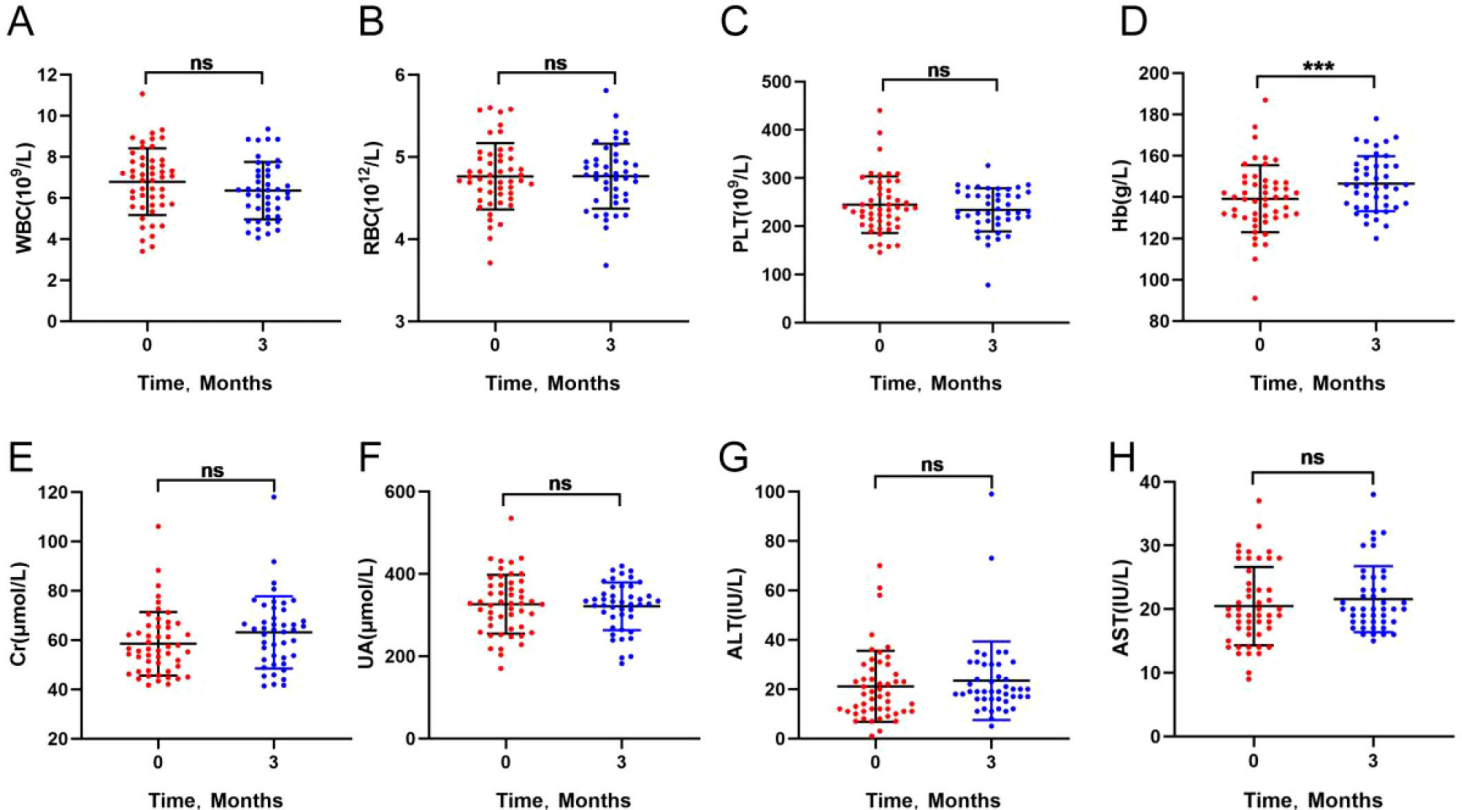
Changes in clinical safety indicators before and 12 weeks after IL-17A inhibition **(A)** WBC, **(B)** RBC, **(C)** PLT, **(D)** ALT, **(E)** AST, **(F)** Cr, **(G)** UA, **(H)** Hb. Data are shown as individual points with median and IQR. ns, not significant; *p* < 0.001 (paired t-test). WBC, white blood cell; RBC, red blood cell; PLT, platelet; ALT, alanine aminotransferase; AST, aspartate aminotransferase; Cr, creatinine; UA, uric acid; Hb, hemoglobin.

Clinical safety indicators before and 12 weeks after IL-17A inhibition showed no significant difference, including white blood cell count (6.79 ± 1.62 vs. 6.35 ± 1.40 x 10^9^/L, p = 0.10; **Figure 3A**), red blood cell count (4.77 ± 0.40 vs. 4.77 ± 0.39 x 10¹²/L, p = 0.81; **Figure 3B**), platelet count (244.70 ± 58.79 vs. 234.10 ± 44.78 x 10^9^/L, p = 0.69; **Figure 3C**), ALT (21.14 ± 14.39 vs. 23.42 ± 15.86 U/L, p = 0.34; **Figure 3D**), AST (20.47 ± 6.17 vs. 21.57 ± 5.19 U/L, p = 0.22; **Figure 3E**), creatinine (58.52 ± 12.88 vs. 63.09 ± 14.67 μmol/L, p = 0.11; **Figure 3F**), and serum uric acid (326.30 ± 71.58 vs. 321.20 ± 58.04 μmol/L, p = 0.76; **Figure 3G**). Hemoglobin increased significantly (139.20 ± 16.20 vs. 146.50 ± 13.30 g/L, *p* < 0.001; **Figure 3H**). No adverse events were reported.

### 3.4 IL-17A inhibition reduced blood pressure in patients with ax-SpA

**Figure 4:**
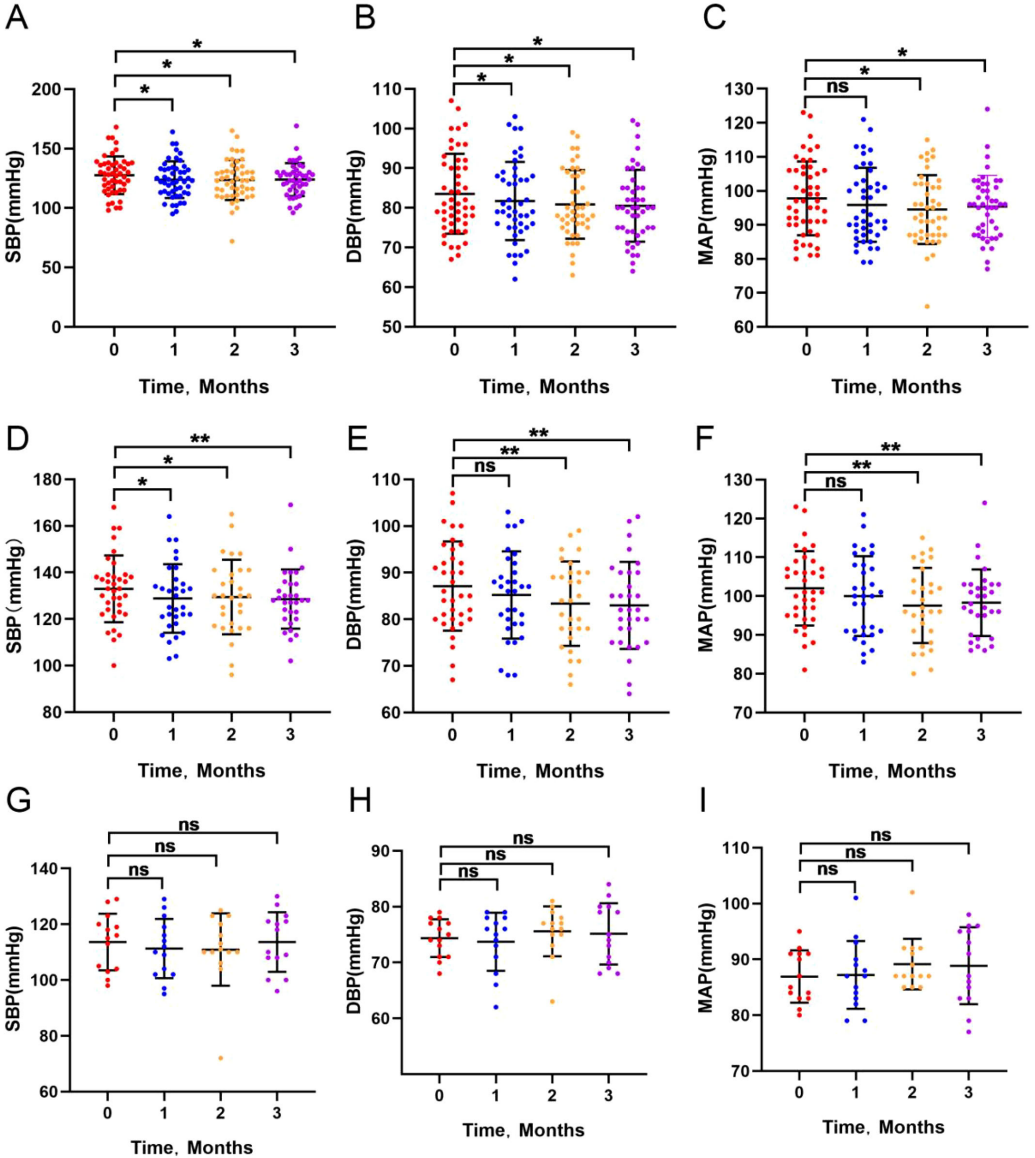
Changes in blood pressure before and during IL-17A monoclonal antibody treatment. **(A-C)** all patients: SBP, DBP, MAP. **(D-F)** hypertensive subgroup. **(G-I)** normotensive subgroup. Data are shown as individual points with median and IQR. SBP, systolic blood pressure; DBP, diastolic blood pressure; MAP, mean arterial pressure. ns, not significant; *p < 0.05, **p < 0.01 (paired t-test).

Blood pressure analysis in all the subjects showed a steady downward trend. After 4 weeks, SBP decreased from 127.60 ± 15.83 mmHg to 123.80 ± 15.39 mmHg (Δ = -3.87 ± 10.31 mmHg, *p =* 0.0125; **Figure 4A**), DBP from 83.54 ± 10.09 mmHg to 81.72 ± 9.84 mmHg (Δ = -2.13 ± 7.17 mmHg, p = 0.05; **Figure 4B**), and MAP from 97.78 ± 10.87 mmHg to 95.86 ± 10.89 mmHg (Δ = -1.57 ± 8.25 mmHg, p = 0.2139; **Figure 4C**). At 12 weeks, reductions were more pronounced: SBP to 123.9 ± 13.87 mmHg (Δ = -3.31 ± 9.05 mmHg, *p* = 0.0181, **Figure 4D**), DBP to 80.53 ± 9.04 mmHg (Δ = -3.27 ± 8.32 mmHg, *p* = 0.0116; **Figure 4E**), and MAP to 95.38 ± 9.15 mmHg (Δ = -2.40 ± 7.38 mmHg, *p* < 0.0345, **Figure 4F**).

After 12 weeks, the levels of SBP, DBP, and MAP also reduced significantly: SBP from 133.00 ± 14.31 mmHg to 128.60 ± 12.68 mmHg (Δ = -4.81 ± 9.35 mmHg, *p* = 0.0076; **Figure 4D**), DBP from 87.11 ± 9.56 mmHg to 82.97 ± 9.34 mmHg (Δ = -5.10 ± 8.38 mmHg, *p* = 0.002; **Figure 4E**), and MAP from 102.00 ± 9.58 mmHg to 98.32 ± 8.57 mmHg (Δ = -4.36 ± 7.06 mmHg, *p* = 0.0018; **Figure 4F**), respectively. However, no significant changes were observed in the normal blood pressure group (**Figures 4G–I**). Furthermore, 7 of the 36 (19.4%) hypertensive patients achieved normal range of blood pressure (SBP *<* 130 mmHg and DBP *<* 80 mmHg).

### 3.5 Linear mixed model analysis of blood pressure changes

A linear mixed-effects model was employed to analyze changes in blood pressure over time, adjusted for age, gender, concomitant medication use, and baseline hypertension status, the results were shown in **Table 2**. Compared to the hypertensive group, the normotensive group had a 14.89 mmHg lower systolic blood pressure (95% CI: -22.36, -7.42, p < 0.001) and a 9.41 mmHg lower diastolic blood pressure (95% CI: -13.67, -5.15, *p* < 0.001). This substantial difference validates the baseline hypertension definition and underscores the necessity of strict adjustment for disease status in the analysis. DBP decreased over time (β = -0.91 mmHg/month, 95% CI: -1.64 to -0.17, *p* = 0.016), while SBP also showed a declining trend (β = -0.90 mmHg/month, 95% CI: -1.87 to 0.07, *p* = 0.071). The effects of age on both systolic and diastolic blood pressure did not reach statistical significance (*p* = 0.160 and 0.065, respectively). Gender, use of nonsteroidal anti-inflammatory drugs, and use of antihypertensive medications showed no significant effects on blood pressure (all *p* > 0.05).

**Table 2:** Results of the linear mixed-effects model for blood pressure changes.

Variable	SBP β (95% CI)	*P* value	DBP β (95% CI)	*P* value
Time (4 weeks)	-0.90 (-1.87, 0.07)	0.071	-0.91 (-1.64, -0.17)	0.016
Age(year)	0.17 (-0.06, 0.40)	0.160	-0.13 (-0.26, 0.01)	0.065
Gender (male)	2.44 (-4.49, 9.37)	0.493	0.11 (-3.87, 4.09)	0.956
NSAID (yes)	1.56 (-5.18, 8.30)	0.652	2.30 (-1.57, 6.17)	0.251
Hypertension status (no)	-14.89 (-22.36, -7.42)	<0.001	-9.41 (-13.67, -5.15)	<0.001
Antihypertensive drugs(yes)	3.55 (-6.01, 13.11)	0.466	3.90 (-1.56, 9.36)	0.167

Further analysis revealed that the interaction between time and antihypertensive medication use was statistically significant for diastolic blood pressure (χ² = 7.487, *p* = 0.024), while marginally significant for systolic blood pressure (χ² = 4.831, *p* = 0.089).

To assess the robustness of the findings, a series of sensitivity analyses were conducted. First, comparisons of different random-effects structures indicated that the random-intercept model adequately fitted the data. Second, among various covariate combinations, the full model indicated the best fit. Finally, we examined the consistency of the results under two different hypertension diagnostic criteria. As presented in **Table 3**, DBP decreased significantly over time under both the ACC/AHA (≥130/80 mmHg) and ESC/ESH (≥140/90 mmHg) criteria, and SBP also showed a declining trend. Sensitivity analysis of time effects indicated that the main conclusions were not changed by choice of hypertension diagnostic threshold.

**Table 3:** Sensitivity analysis of time effects under different definitions of hypertension.

Variable	SBP β (SE)	*P* value	DBP β (SE)	*P* value
ACC/AHA (≥130/80 mmHg)	-0.898 (0.494)	0.071	-0.909 (0.372)	0.016
ESC/ESH (≥140/90 mmHg)	-1.005 (0.498)	0.044	-1.036 (0.375)	0.006

## 4 Discussion

This exploratory pilot study suggests that IL-17A inhibition significantly decreased disease activity and systemic inflammation in patients with ax-SpA. After 12 weeks, ASDAS-CRP and ASDAS-ESR scores had decreased markedly. Inflammatory levels (CRP, ESR, IL-6) were also significantly reduced. The observed increase in hemoglobin levels may be attributable to inflammation.

The treatment indicated a favorable safety profile, with stable hematological, hepatic, and renal parameters and no hypotension-related adverse events. Importantly, a longitudinal decline in blood pressure was observed over the treatment time. Linear mixed-effects model analysis, after adjusting for relevant confounders, showed a mean reduction of 0.90 mmHg in SBP every 4 weeks (*p*= 0.071) and a reduction of 0.91 mmHg in DBP every 4 weeks (*p*= 0.016). These findings remained robust in sensitivity analyses. Overall, the results indicate a longitudinal association between IL-17A inhibition and blood pressure lowering, particularly for diastolic blood pressure in patients with hypertension, though causality cannot be inferred due to the uncontrolled, single-arm design of this exploratory study.

TNF-α inhibitors have been associated with reduced cardiovascular events (17). This study indicated the potential BP-lowering effects of IL-17A inhibition and the involved mechanisms might be different. IL-17A has been shown to inhibit endothelial nitric oxide synthase (eNOS), promote vascular remodeling (18, 19), and upregulate renal sodium transporters, contributing to volume expansion (20). IL-17A-induced renal inflammation and fibrosis may impair glomerular function and activate the renin-angiotensin system (RAS) (21). Furthermore, IL-17A may compromise blood-brain barrier integrity, facilitating central sympathetic activation and neurogenic hypertension (22, 23). Thus, IL-17A blockade may improve hypertension through multiple pathways. Neuroinflammation in the paraventricular nucleus of the hypothalamus has been implicated in the pathogenesis of hypertension. Mouse models may provide an opportunity to contextualise the obtained data (24).

Patients with AS face an elevated cardiovascular risk attributable to chronic systemic inflammation, comorbidities (e.g., hypertension, diabetes, dyslipidemia), and potential side effects of long-term use of NSAIDs (25–29). Chronic inflammation accelerates atherosclerosis (30), whereas NSAIDs may further increase cardiovascular risk (31). Biological agents such as TNF-α inhibitors, and potentially IL-17A inhibitors may mitigate this risk through their anti-inflammatory effects (32, 33).

We hypothesize that IL-17A blockade may enhance the antihypertensive effects of RAAS inhibitors by attenuating angiotensin II-induced IL-17 production and subsequent vascular inflammation—precisely the “dual effect” proposed by Deussen et al (34).

However, several limitations must be acknowledged. Owing to the uncontrolled, single-arm design, causality cannot be inferred from the observed associations. The present findings should therefore be regarded as hypothesis-generating observation and require validation through randomized controlled trials. The absence of data on IL-17A levels at baseline and during treatment precludes confirmation of IL-17A alteration and investigation of whether blood pressure changes correlated with the degree of IL-17A inhibition. Potential confounding factors (e.g., improved physical activity, unrecorded medication adjustments, regression to the mean) may have influenced the results. Despite adjustments for antihypertensive medication use, residual confounding from unmeasured changes in medication adherence or lifestyle factors cannot be excluded. The small sample size and 12-week follow-up is relatively short and cannot assess the durability of the BP-lowering effect or its impact on hard cardiovascular endpoints. Despite these limitations, the association between reduced inflammation and improved blood pressure control supports further investigation of IL-17A-targeted therapies for cardiovascular risk modification in ax-SpA. Future studies should employ randomized controlled designs with extended follow-up, active comparators, systematic medication tracking, and predefined cardiovascular endpoints.

In conclusion, this exploratory study provides preliminary evidence that IL-17A inhibition is associated with improved disease status and a concomitant, time-dependent reduction in blood pressure in patients with AS, with a more rapid decline in DBP in those with baseline hypertension. These findings highlight the need to integrate cardiovascular outcome research in ax-SpA into the broader investigation of autoimmune diseases. Definitive conclusions await validation in randomized controlled trials.

## Data Availability

The raw data supporting the conclusions of this article will be made available by the authors, without undue reservation.
